# Conspiracy mentality, subclinical paranoia, and political conservatism are associated with perceived status threat

**DOI:** 10.1371/journal.pone.0293930

**Published:** 2023-11-22

**Authors:** William N. Koller, Honor Thompson, Tyrone D. Cannon

**Affiliations:** 1 Department of Psychology, Yale University New Haven, Connecticut, United States of America; 2 Department of Psychiatry, Yale University New Haven, Connecticut, United States of America; Yeditepe University, TURKEY

## Abstract

Status threat (i.e., concern that one’s dominant social group will be undermined by outsiders) is a significant factor in current United States politics. While demographic factors such as race (e.g., Whiteness) and political affiliation (e.g., conservatism) tend to be associated with heightened levels of status threat, its psychological facets have yet to be fully characterized. Informed by a “paranoid” model of American politics, we explored a suite of possible psychological and demographic associates of perceived status threat, including race/ethnicity, political conservatism, analytic thinking, magical ideation, subclinical paranoia, and conspiracy mentality. In a small, quota sample drawn from the United States (N = 300), we found that conspiracy mentality, subclinical paranoia, conservatism, and age were each positively (and uniquely) associated with status threat. In addition to replicating past work linking conservatism to status threat, this study identifies subclinical paranoia and conspiracy mentality as novel psychological associates of status threat. These findings pave the way for future research regarding how and why status threat concerns may become exaggerated in certain individuals, possibly to the detriment of personal and societal wellbeing.

## Introduction

In his classic 1964 essay, Richard Hofstadter described the American political landscape as “paranoid,” noting that "no other word adequately evokes the sense of heated exaggeration, suspiciousness, and conspiratorial fantasy" that characterized United States (U.S.) politics at the time [1, p. 1]. A consistent feature of this “paranoid style” is the perpetual promise of threat to the American people, the purported source of which has ranged from the Freemasons in the late 18th century to Communists throughout the 20th century. Today, a new body of research has emerged that organizes this genre of unease using the concept of *perceived status threat*, which denotes concern that power derived from membership to a dominant social group will be challenged by out-group members–especially by racial minorities (for reviews, see [2, 3]).

Status threat is thought to be an important factor in current U.S. politics, especially among White Americans, who represent the current majority racial group in the U.S. [4]. For example, when the possibility of an upcoming racial majority-minority shift is made salient, White Americans tend to endorse more conservative policy positions [5, 6]. Stronger identification with White racial identity has also been associated with greater nostalgia for a time when White Americans enjoyed unchallenged status–and this “racial nostalgia” is in turn linked to greater support of White nationalist ideologies (e.g., “In order to maintain White status it is sometimes necessary to use violence towards racial/ethnic minority groups”) [7]. The language of status threat has been readily adopted by some conservative movements, who point to the socioeconomic elite and racial minorities (immigrants, in particular) as a cause of the American people’s woes [8]. Accordingly, a popular theme in modern American right-wing populism is that those both lower and higher in a perceived social hierarchy are working together to undermine a certain class or race [9, 10], and politically conservative White participants have been found to endorse high levels of group status threat at baseline (i.e., even without exposure to information about an impending racial shift [11].

Yet despite the importance of status threat in American politics, its psychological correlates are not well understood. To this end, we sought to characterize the interrelationships between perceived status threat and a suite of psychological variables, above and beyond likely demographic associates (e.g., political affiliation and race/ethnicity). In particular, we focus on psychological factors that may be particularly relevant to Hofstadter’s “paranoid style” of American politics, including conspiracy mentality, subclinical paranoia, magical ideation, and engagement in analytic thinking, which we review in turn below.

### Conspiracy mentality

First, we anticipate that perceived status threat may be connected to conspiracy mentality–defined as the tendency to endorse explanations of significant events as the result of the covert action of malevolent groups [12]. In line with this notion, “socio-epistemic” models of conspiracy mentality suggest that mistrust of out-groups and major institutions are central to this type of thinking [13]. Often emerging in times of societal or political upheaval during which in-group society appears threatened, these “inter-group” conspiracy theories may help maintain a sense of control by attributing the source of threat to minority groups [14]. Accordingly, inter-group conspiracy theories have been found to foster prejudice against out-group members who become scapegoats for perceived threats to in-group security [15]. These themes are evident in conspiracy theories that are currently popular in the U.S. For instance, the “QAnon” family of conspiracy theories focuses on an imminent threat to a subset of the American people (i.e., “patriots”) at the hands of corrupt elites and other purportedly nefarious out-group members (e.g., immigrants, Jewish people) [16]. In line with these themes, text analyses of posts made by “Q” (whose posts on message boards such as 4chan, 8chan, and 8kun popularized the QAnon movement) reveal recurring references to group oppression and marginalization as a call to action (e.g., “The time has come to take back our great land) [17]. Together, this literature supports a strong connection between status threat and conspiracy mentality, indicating that mistrust of out-group members and concern about group marginalization may be closely tied to conspiracy theorizing in the U.S.

### Subclinical paranoia

The connection between status threat and paranoia can be considered in light of a “coalitional” account of paranoia, which suggests that persecutory ideation arises from evolved social cognitive mechanisms focused on managing inter-group conflict in competitive environments [18]. According to this model, paranoia is most likely to arise in the context of *coalitional threats–*challenges posed by an out-group to one’s evolutionary fitness. In support of this hypothesis, belonging to a marginalized social group or experiencing discrimination (i.e., both forms of coalitional threat) is associated with paranoia among those at high-risk for psychosis [19] and in the general population [20]. Yet at the other end of the spectrum of status, this model might predict similarly elevated paranoia among members of *higher* status social groups–if, that is, they were concerned that this status was at risk. This is consistent with empirical work suggesting a U-shaped relationship between subjective social status and paranoia: individuals who estimated themselves to be at the lowest and highest rungs of social status reported higher paranoia than those in the middle [21]. This same study found that interacting with an individual from a higher social status or political out-group (i.e., inducing coalitional threat) increased ratings of paranoia. This relationship between social threat and paranoia is further supported by a study which used a virtual reality paradigm to manipulate participants’ in-game heights as a proxy for social status [22]: here, *reducing* a participant’s height relative to other characters in the virtual environment (i.e., reducing social status) *increased* subjective reports of paranoia.

Given that status threat entails concern about out-group members undermining in-group security, this literature would suggest that perceived status threat may arise from similar social cognitive processes (i.e., those that concern the detection of coalitional threat) that, at their extreme, also engender paranoia. Conversely, social threat (of which status threat may be one dimension) seems to induce paranoia. Given this potentially reciprocal relationship, we suggest that status threat is likely closely interrelated with subclinical experiences of paranoia in daily life (as either a cause or a consequence). Importantly, while conspiracy mentality can involve paranoid themes, in that it frequently touches on interpersonal or coalitional threats, these constructs have been found to be distinct (yet positively correlated) [23, 24]. This suggests that, despite their conceptual overlap, paranoia and conspiracy mentality may account for *unique* variance in perceived status threat.

### Magical ideation

Beyond paranoia, might there be other aspects of odd or psychotic-like thought (to which paranoia is closely related) [25, 26] that are related to perceived status threat? To address this question, we additionally sought to assess whether status threat is associated with *magical thinking*, which can be defined as the tendency to believe that objectively unrelated occurrences are causally linked [27]. Experimentally, magical ideation is associated with illusory pattern perception [28] and greater acceptance of illusory contingencies [29]. Magical ideation is further associated with belief in conspiracy theories [30, 31], which often focus on tenuous causal connections between the behavior of out-group members and in-group wellbeing (as reviewed above). As such, magical ideation could conceivably represent a correlate of status threat insofar as these concerns involve the uncritical endorsement of weakly related events as being causally connected (e.g., believing that changes in one’s personal wellbeing is linked to increasing numbers of racial minorities in America, even if there is limited evidence for this causal association). Notably, magical ideation is more domain-general than paranoia–i.e., it is not necessarily related to concerns with interpersonal threat. In this way, including a measure of magical ideation in the present study helps us discern whether status threat is more closely connected to differences in causal thinking writ large versus more specific concerns with interpersonal threat *per se* (i.e., as measured by paranoia).

### Analytic thinking

Finally, we surmised that analytic thinking–typically defined as the capacity to override an intuitive impression using more deliberative thinking [32]–could be associated with status threat. While this study is the first to our knowledge to measure this association, we can draw from related literatures to inform our expectations. For instance, analytic thinking has recently received a good deal of attention as a potential driver of engagement with “fake news”. A growing body of work suggests that belief in fake news headlines is associated with the tendency to engage in analytic thinking [33, 34], such that people who tend to rely on an initial intuitive impression (versus engage in more deliberative reasoning) are more likely to believe false headlines. Importantly, fake news itself is often deeply connected with issues of status threat in that many of the most provocative and influential articles in this genre pertain to a sense of threat to a particular demographic [35]. Analytic thinking has also been closely linked to belief in conspiracy theories [36]. As such, reduced engagement in analytic thinking could represent a plausible pathway via which status threat concerns take root or are maintained, potentially due to greater uncritical acceptance of high-valence but low-veracity information found in highly partisan, conspiratorial, or low-quality sources.

### Present study

Based on the foregoing, we conducted exploratory analyses in a quota sample collected using the Prolific platform to determine whether this suite of psychological constructs (subclinical paranoia, magical ideation, analytic thinking, and conspiracy mentality) was associated with perceived status threat, above and beyond demographic factors such as political affiliation (e.g., conservatism) and race (e.g., Whiteness). Importantly, characterizing the psychological factors that co-occur with perceived status threat may pave the way for new insights regarding what leads people to endorse these views to extreme degrees, thereby helping us to better understand the processes via which status threat interacts not only with America’s politics but also with the psychological wellbeing of its people.

## Methods

### Preregistered analyses

Note that the data reported herein were collected in the service of an undergraduate thesis at Yale University, in accordance with a preregistered data collection and analysis plan (see https://aspredicted.org/G75_1J8). This manuscript reports exploratory, non-preregistered analyses using this same data. For completeness’ sake, the preregistered analyses from the thesis project are reported in full in the S1 File. Notably, while those analyses revealed interrelationships between status threat and both subclinical paranoia and conspiracy mentality (as characterized in more detail below), they suggested that status threat *does not* moderate relationships between political extremism and paranoia, magical ideation, or conspiracy mentality (see Section 1 of S1 File for more detail).

### Participants

Data were collected from 300 participants on Prolific (prolific.co) in March 2022 using a brief Qualtrics questionnaire in exchange for a payment of $5 (representing a final rate of $23.11 per hour). Only participants who were over the age of 18 and who were located in the U.S. were recruited. Importantly, we used Prolific’s quota sampling tool to ensure that age, sex, and race/ethnicity closely matched the proportions found in the U.S. population (see https://researcher-help.prolific.co/hc/en-gb/articles/360019236753-Representative-samples for more details). Note that while we initially preregistered a final sample of 250 participants, we deviated from our data collection plan in order to satisfy the minimum number of participants required by this quota sampling tool (N = 300). All participants passed three attention check questions randomly distributed throughout the questionnaire (e.g., “Please select “Strongly Agree (7) from the options below”); hence, no participants were excluded from the final sample. Participants included 145 cisgender men, 153 cisgender women, 1 transgender woman, and 1 non-binary individual. Average age was 45.01 (*SD* = 15.87). 205 (68.33%) participants identified as White, 44 (14.67%) as Black, 25 (8.33%) as Asian, 15 (5.00%) as Multiracial, 1 (0.03%) as Native American, and 10 (3.33%) selected “Other” for the question of race. In terms of political affiliation, 177 (59.00%) participants identified as liberal, 78 (26.00%) as conservative, and 45 (15.00%) as moderate. See Table 1 of S1 File for more details on demographic information.

### Measures

Perceived status threat (ST) was measured using items designed to index Americans’ resistance to social and racial change, as used in prior studies [11]. Participants were asked to indicate how much they agreed with eight statements about the future of America and the influence of minorities on their wellbeing (e.g., “Compared to today, 50 years from now what it means to be a true American will be less clear”; “Americans should be alarmed that racial minorities are representing an increasingly large proportion of the U.S. population”) on a scale of 1 (Strongly Disagree) to 7 (Strongly Agree). The responses to these questions were then summed (ranging from 0 to 56), with higher scores indicating greater endorsement of status threat.

Analytic thinking was measured using a seven-item Cognitive Reflection Test (CRT) that included questions from the original three-item CRT [37] as well as a version that relies less on math abilities [38]. The CRT is comprised of questions that require participants to override an initial intuitive but incorrect response to come to a correct answer (e.g., “How many cubic feet of dirt are there in a hole that is 3’ deep x 3’ wide x 3’ long?”; answer = 0 cubic feet). Performance on the CRT was indexed by the sum of correct answers (ranging from 0 to 7), with higher scores indicating greater engagement in analytic thinking.

Subclinical paranoia was measured using the Revised Green Paranoid Thoughts Scale, part B (R-GPTS-B) [39]. The R-GPTS-B consists of 10 items which query thoughts and feelings one may have had about others in the past month (e.g., “I was distressed by being persecuted”). Participants were instructed to indicate the extent to which they experienced these thoughts and feelings on a scale of 0 (Not at All) to 4 (Totally). Paranoid ideation was indexed by the sum of a participant’s responses (ranging from 0 to 40), with higher scores indicating higher levels of paranoia.

Tendency to engage in magical thinking was measured via the Magical Ideation Scale (MIS) [27]. In the MIS, participants are asked to respond with either “True” or “False” to 30 assertions about superstitious or paranormal phenomena (e.g., “Numbers like 13 and 7 have no special powers”; “I have sometimes sensed an evil presence around me, although I could not see it”). Participants’ responses were then coded to indicate whether their answer indicated engagement in magical thinking (1 = magical thinking, 0 = no magical thinking) and summed to create a final score (ranging from 0 to 30), with higher scores indicating higher levels of magical ideation.

Conspiracy mentality was measured using the Conspiracy Mentality Questionnaire (CMQ) [40], which is composed of five statements designed to index general susceptibility to conspiratorial thinking (as opposed to the endorsement of specific conspiracy theories). The CMQ includes statements such as “I think that government agencies closely monitor all citizens” and “I think that events which superficially seem to lack a connection are often the result of secret activities.” Participants were asked to indicate the extent to which they believe each statement, from 0 (Certainly Not) to 10 (100% Certain). Each participant’s answers were summed to create a final score (ranging from 0 to 50), with higher scores indicating greater susceptibility to conspiracy mentality.

Finally, political affiliation was assessed using a seven-point scale (1—Extremely Liberal, 2—Somewhat Liberal, 3—Slightly Liberal, 4—Moderate, 5—Slightly Conservative, 6—Somewhat Conservative, 7—Extremely Conservative). This scale was adapted from prior studies on perceived status threat [11], with two additional options added for greater precision.

The internal consistencies of all questionnaire measures were indexed using Omega total [41]. This metric is the result of a factor analysis of all items on a scale, followed by an oblique rotation and extraction of a general factor. It can be interpreted using similar cut-offs as Cronbach’s alpha (i.e., a value of 0.9 reflecting excellent internal consistency). Descriptive statistics and Omega Total for these measures can be found in Table 1. All scales exhibited acceptable to excellent internal consistency. These scales have also been reported to demonstrate excellent face, convergent, discriminant and/or predictive validity (see original publications); for the status threat measure, which was compiled from several sources [11], see also references [6, 42–44].

**Table 1:** Descriptive statistics and omega total questionnaire measures.

Questionnaire	Mean (*SD*)	Omega Total (ω_t_)
R-GPTS-B	4.85 (6.77)	ω_t_ = 0.92
CRT	3.81 (1.61)	ω_t_ = 0.82
MIS	5.19 (4.52)	ω_t_ = 0.85
ST	26.87 (7.95)	ω_t_ = 0.80
CMQ	32.95 (11.75)	ω_t_ = 0.90
Political Affiliation	3.25 (1.89)	N/A

### Procedure

This study’s protocol (2000026576) was deemed exempt by the Yale University IRB. After obtaining written consent, we asked participants to respond to a series of questionnaires via Qualtrics, presented in counterbalanced order and including semi-randomly dispersed attention check questions. Demographic information was subsequently collected.

### Analyses

All analyses were conducted in R. Outliers in non-skewed data were defined as points greater than 3 *SD*s from the sample mean; outliers in skewed data were defined using robust measures [45], as implemented by the RobustBase package [46]. Identified outliers in questionnaire measures were winsorized [47], preserving rank order.

We used Akeike’s Information Criteria (AIC) [48] to conduct stepwise regression (both backwards and forwards) via R’s “step” command, distinguishing between a set of possible multiple linear regression models describing the relationships between status threat and a number of demographic (i.e., race/ethnicity, political affiliation, age, gender, socioeconomic status, and education) and psychological factors (i.e., conspiracy mentality, subclinical paranoia, analytic thinking, and magical ideation). Independent variables whose inclusion failed to reduce model AIC were excluded from the final model. Finally, we additionally conducted a partial correlation analysis to further characterize the covariance structure of surviving and statistically significant variables of interest using the R’s ‘ppcor’ package [49].

## Results

Zero-order correlations between variables of interest can be found in Table 2.

**Table 2:** Zero-order correlations between variables of interest.

	2	3	4	5	6
1. R-GPTS-B	-.02	-.13*	.52***	.25***	.33***
2. Political Affiliation	—	-.07	.02	.25***	.32***
3. CRT	—	—	-.22***	-.24***	-.05
4. MIS	—	—	—	.50***	.28***
5. CMQ	—	—	—	—	.44***
6. ST	—	—	—	—	—

*Note*. Non-parametric correlations (Spearman’s rho) are reported as variable distributions were non-normal.

*: *p* < .05,

**: *p* < .01,

***: *p* < .001.

### Stepwise regression

Our final model was composed of the demographic and psychological variables that comprised the best fitting model according to AIC. These included CMQ score, R-GPTS-B score, political affiliation, CRT score, ethnicity, and age (final model AIC = 1999). All other independent variables (race, gender, level of education, and MIS score) were excluded as they did not improve model fit. We used the vif function of the car package [50] to test whether the data met the assumption of collinearity; this analysis indicated that multicollinearity was not a concern (all VIFs < 1.3).

**Fig 1:**
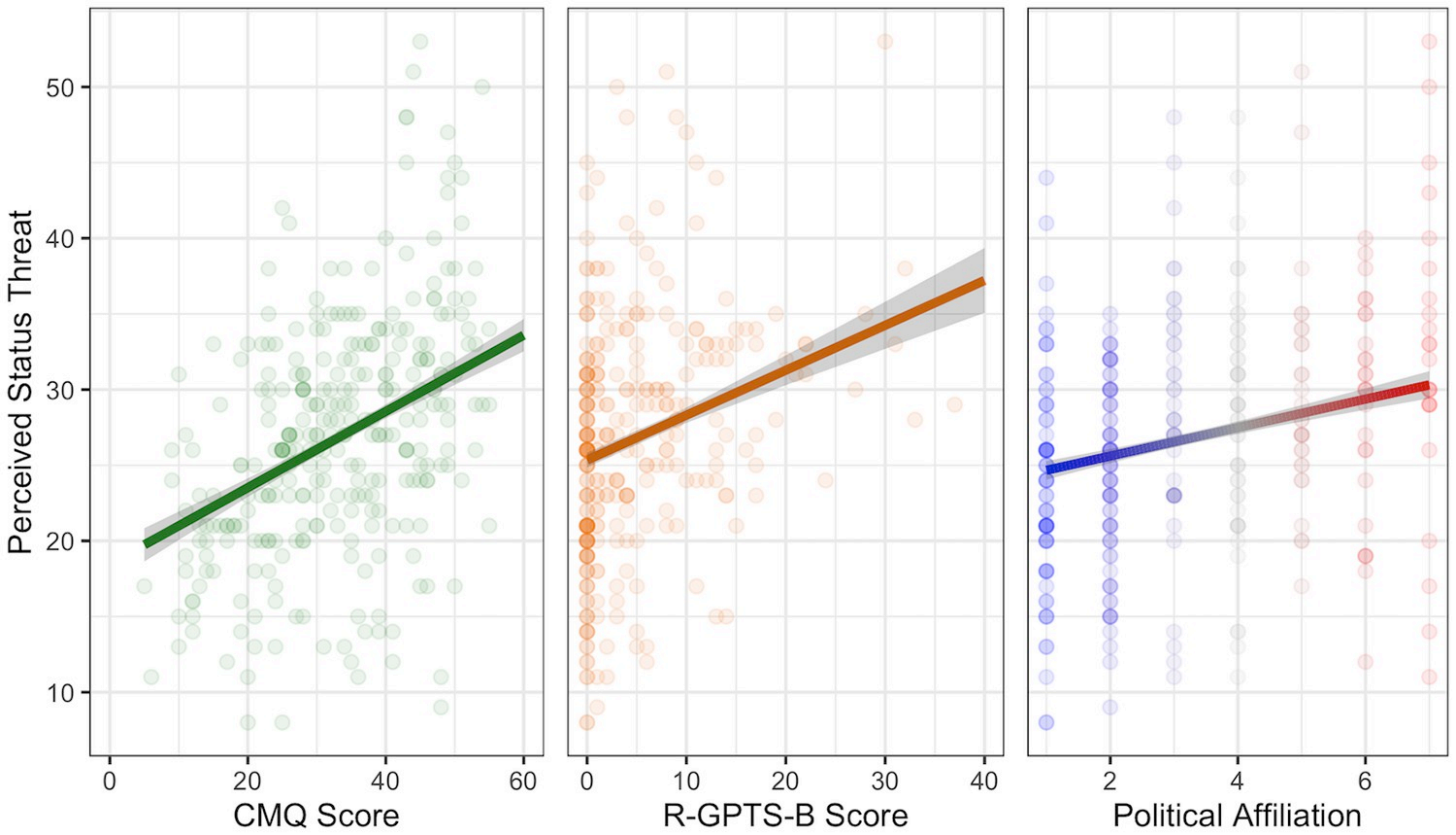
Perceived status threat as a function of conspiracy mentality (CMQ score), subclinical paranoia (R-GPTS-B score), and political affiliation. *Note*: Shaded area represents *SE*. Political affiliation ranges from 1 (Very Liberal) to 7 (Very Conservative).

Collectively, these variables accounted for 30% of the variance in status threat, *F*(6, 293) = 21.90, *p* < .001, *R*^2^_Adj._ = 0.30. In line with our literature review, CMQ score was significantly positively associated with ST score, *ß* = 0.37, 95% CI [0.26, 0.48], *p* < .001, such that participants who endorsed higher levels of conspiracy mentality also tended to report greater degrees of status threat (see left panel of Fig 1). ST score was also significantly positively associated with both R-GPTS-B score, *ß* = 0.25, 95% CI [0.15, 0.35], *p* < .001, and political affiliation, *ß* = 0.22, 95% CI [0.12, 0.33], *p* < .001 (see middle and right panels of Fig 1, respectively). Here, participants who endorsed higher levels of subclinical paranoia and participants who identified as more conservative, respectively, both tended to report greater degrees of status threat. Finally, there was a significant effect of age, *ß* = 0.11, 95% CI [0.01, 0.21], *p* < .03, such that older participants tended to report greater degrees of status threat. Surprisingly, no significant effects emerged for ethnicity, *ß* = -0.20, 95% CI [-0.62, 0.21], *p* = .34, or CRT score, *ß* = 0.09, 95% CI [-0.01, 0.19], *p* = .07. Similarly, neither race nor magical ideation met criteria for inclusion in the final model, indicating that these factors did not uniquely account for variance in status threat above and beyond conspiracy mentality, subclinical paranoia, political affiliation, age, and analytical thinking.

### Partial correlation analysis

**Fig 2:**
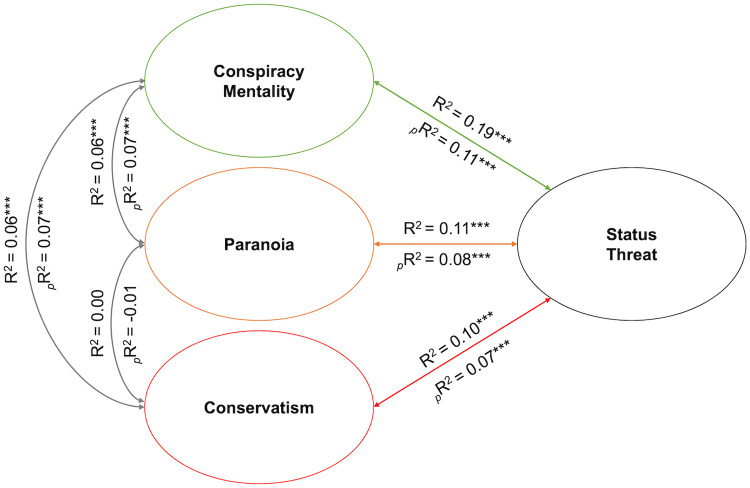
Partial correlation analysis results. *Note*: Coefficients above the line represent first-order correlations; those below the line represent partial correlations. *: *p* < .05, **: *p* < .01, ***: *p* < .001.

Partial correlation analysis revealed that CMQ score accounted for 11% of the variance in ST score when covarying for both R-GPTS-B score and political affiliation, _*p*_R^2^ = 0.11, *p* < .001. R-GPTS-B score accounted for 8% of the variance in ST score when covarying for CMQ score and political affiliation, _*p*_R^2^ = 0.08, *p* < .001. Finally, political affiliation (i.e., conservatism) accounted for 7% of the variance in ST score when covarying for CMQ and R-GPTS-B score, _*p*_R^2^ = 0.07, *p* < .001. These relationships are depicted in Fig 2, with first-order correlation coefficients appearing above the arrows and partial correlation coefficients appearing below. Interestingly, contrasting first-order with partial correlation coefficients revealed minimal overlap *between* constructs of interest in their association with ST score: R-GPTS-B score and political affiliation each shared only 3% of the variance in ST score with all other variables, while CMQ score shared 8% of the variance in ST score with all other variables. Further, this analysis failed to reveal an association between subclinical paranoia and conservatism, _*p*_R^2^ = -0.01, *p* = .13. Thus, while each construct was associated with ST score, they accounted for largely unique portions of its variance.

## Discussion

In the present study, we explored a novel suite of psychological factors that we posited may be particularly relevant to a longstanding “paranoid style” of American politics [1]. Our analyses revealed that conspiracy mentality and subclinical paranoia, in addition to political affiliation and age, were each significantly and uniquely associated with perceived status threat. Namely, participants who expressed greater propensity for conspiracy mentality, endorsed higher degrees of paranoia, reported being more conservative, and/or were older also tended to endorse greater degrees of status threat. Notably, partial correlation analyses revealed minimal overlap between the variance accounted for by paranoia, conspiracy mentality, and conservatism: they appeared to be largely orthogonal, rather than overlapping or interactive (see also Section 1 of S1 File). In other words, those who reported elevated paranoia and are concerned with status threat were *not* necessarily be the same people who were politically conservative and concerned with status threat. This suggests that these constructs could be involved in largely parallel pathways via which status threat concerns may manifest. Together, these results carry several important implications both for our understanding of the construct of status threat and its interrelationships with demographic and psychological factors, as discussed below.

### Conspiracy mindset and subclinical paranoia

The results of the present study indicate that both conspiracy mentality and subclinical paranoia represent important psychological associates of perceived status threat, with their respective terms explaining a greater (in the case of conspiracy mentality) or similar (in the case of paranoia) amount of variance in the model as conservatism. In this way, this study broadens the discussion around status threat to include novel psychological variables that may be related to the genesis or maintenance of these concerns, above and beyond political affiliation.

This study also bolsters our understanding of conspiracy mentality. While themes of in-group marginalization appear to be popular in modern conspiracy theories [17], our results help to more formally establish this connection. This work invites future longitudinal research aimed at teasing apart the directionality of this association, which remains unclear. One possibility is that susceptibility to conspiracy mentality may precede and subsequently promote status threat concerns, whereby one becomes increasingly likely to attribute sociopolitical phenomena (e.g., a perceived loss of power) to the actions of “nefarious” groups (e.g., minorities, immigrants). This is consistent with work showing that stronger belief in “minority collusion” (the idea that minorities form a unified political bloc that acts against in-group interest) precedes and predicts support of the White nationalist “Alt-Right” movement, which claims to resist the perceived marginalization of White Americans [51]. On the other hand, underlying feelings of status threat could lead people to subsequently seek confirmation of these concerns from low-quality sources, leading down a “rabbit hole” of conspiracy theories that offer organizing frameworks and engaged communities surrounding issues of in-group marginalization. This notion is supported by work suggesting that inter-group conspiracy theories emerge as a means of reestablishing control following challenges to in-group society [13, 14]. Understanding the nuances of the relationship between conspiracy mentality and status threat is of the upmost importance insofar as conspiracy theories in their most extreme form represent markers of social division and–when fueled by concerns of threat from out-group members–may foment animosity towards specific (and often marginalized) groups [16]. Indeed, conspiracy theories that highlight status threat may be especially compelling as impetuses towards (potentially violent) action against either political elites or racial minorities, as evidenced by the messaging that emerged during the riot at the United States Capitol on January 6^th^ of 2021 [52] and in the language used in the published manifesto of the Buffalo, New York shooter in 2022 (“I am simply a White man seeking to protect and serve my community, my people, my culture, and my race”) [53].

The observed relationship between subclinical paranoia and perceived status threat similarly carries implications for current debates about whether paranoia represents an inherently social versus non-social phenomenon. The present study provides indirect support for a more social “coalitional account” of paranoia, which holds that paranoia may emerge in the context of challenges posed by an out-group to one’s evolutionary fitness [18]. The fact that magical ideation did *not* uniquely contribute to model fit above and beyond paranoia further supports this notion, as it suggests a more discriminant relationship between status threat and paranoia (versus psychotic-like thought writ large). This does not, however, rule out non-social explanations for this relationship. For instance, status threat concerns may manifest as a *result* of psychotic-like experiences rather than the other way around. Here, high degrees of uncertainty generated by the dysfunction of more domain-general (i.e., non-social) processes could eventually get misattributed to the actions of nefarious outsiders, who become a means of explanation for odd subjective experiences (in line with a “non-social” account of paranoia) [54]. An intriguing possibility is that these in fact represent two distinct profiles of or pathways to paranoia. This highlights the importance of understanding the directionality of this relationship, as one pathway (e.g., status threat leading to paranoia) might demand a different intervention strategy than another (e.g., paranoia leading to status threat). Finally, it is possible that the relationship between paranoia and status threat is best characterized as a positive feedback loop: as one becomes more paranoid about others (through either social or non-social processes), one may perceive greater degrees of status threat, which in turn may incite more paranoia, and so on.

### Conservatism and age

By replicating associations between conservatism and status threat, this study lends further support to the idea that the perceived marginalization of “traditional” American values and groups may represent a particular area of concern to politically conservative Americans [51]. Addressing status threat concerns may thus represent an appealing platform for certain subsets of conservative party–a pattern that bears out in recent legislative pushes to suppress the mention of Critical Race Theory (which can be seen by some as marginalizing to White Americans) in classrooms [55]. As with conspiracy mentality and paranoia, however, the direction of the relationship between status threat and conservatism remains unclear: it is possible that status threat concerns promote affiliation with conservatism rather than the other way around. Further, this relationship is far from axiomatic: in the present study, a number of participants who identified as liberal reported higher levels of status threat. Conversely, a number of conservative participants reported lower levels of status threat. As such, while conservatives were more likely to report concern with this issue, perceptions of status threat were not exclusive to those on the political right (consistent with past literature) [5].

This study also revealed a small but positive association between age and perceived status threat, such that older participants tended to endorse greater concern with status threat. The relationship between age and status threat has, to our knowledge, been little discussed. However, we can speculate that this association may be connected to sentiments of lost privilege and nostalgia for the “good old days” [56] that may be particularly palpable to older generations of (White) Americans. It has also been shown that older adults were presented with and shared more fake news during the 2016 U.S. presidential election, with analyses indicating that a greater proportion of political URLs in their newsfeeds originated from fake news sites [57]. Thus, older adults may be more exposed to (and influenced by) highly partisan or exaggerated rhetoric regarding issues of status threat.

### Notable null relationships

It is noteworthy that most demographic variables either did not warrant inclusion in the final model (i.e., race, gender, socioeconomic status, and level of education) or showed null relationships with status threat (i.e., ethnicity). This was particularly surprising for race/ethnicity, given the focus of past literature on status threat among White Americans [2, 3]. What might explain this dissociation? In contrast to our study, which measured status threat at baseline, the seminal work on status threat among White Americans [5, 6] primed participants by having them read an article about impending demographic changes in the U.S. *prior to* assessing status threat. As such, one possibility is that the relationship between Whiteness and status threat is heightened when the idea of a “majority-minority” America is made salient. This would suggest that status threat may not represent a front-of-mind concern for many White Americans–but that these concerns are easily activated by rhetoric focused on out-group incursion. Future research could thus benefit from taking a similar approach to help clarify the nature of the associations between demographic factors such as race and status threat.

Finally, it is worth noting that we observed no statistically significant relationship between political conservatism and paranoia (*r* = -0.02), standing in contrast to research reporting ideological asymmetries in paranoia [58]. This discrepancy may be related to differences in the operationalization of “paranoid ideation”. For example, measuring paranoia using items that are closely related to politically relevant safety concerns (e.g., “Every day, our society becomes more lawless and bestial, a person’s chances of being robbed, assaulted and even murdered go up and up”) [58] may yield stronger associations with conservatism than will more clinical scales like the R-GPTS-B. This highlights the importance of using precise language when interpreting such results: are concerns with lawlessness the same as paranoid ideation in a more psychosis-like sense? How do we distinguish between these constructs using questionnaire measures? These represent important questions for future research that aims to explore the intersection of clinical and political psychology while avoiding the conflation of clinical and sociopolitical constructs.

### Strengths and limitations

To our knowledge, this study represents the first of its kind in that it systematically assessed the unique associations of several key psychological variables with perceived status threat, above and beyond demographic variables. In addition to replicating past work demonstrating an association between status threat and conservatism, this technique revealed novel connections between status threat and both subclinical paranoia and conspiracy mentality. Further, through online data collection, we were able to take advantage of Prolific’s quota sampling, which ensures a group of participants whose demographics reflect the overall population of the U.S.

However, our sample was skewed in terms of political affiliation, in that more participants self-identified as more liberal. This may reflect the underlying demographics of online marketplaces like Prolific, highlighting how online samples may be representative in some ways (e.g., age, sex, race) but not others (e.g., political affiliation). We also used a single-item measure of political affiliation which did not differentiate between social and fiscal conservatism; thus, our measure of conservatism lacks the precision to draw conclusions about certain political subgroups (e.g., libertarians). Further, our conspiracy mentality questionnaire assessed only general susceptibility to this style of thinking; as such, future research could expand upon this study by assessing belief in specific conspiracy theories (e.g., QAnon). Finally, given this study’s cross-sectional design, we cannot speak to causality or directionality in any of the explored relationships. As analyses were exploratory (i.e., not preregistered), they warrant replication in future studies with larger sample sizes; in particular, longitudinal designs could prove useful in testing various possible causal models of the relationships between conservatism, subclinical paranoia, conspiracy mentality, and status threat.

## Conclusion

Almost 60 years ago, Richard Hofstadter described the American political landscape as “paranoid”, based on observations of “heated exaggeration, suspiciousness, and conspiratorial fantasy” [1]. Here, we explored these themes by examining the psychological associates of perceived status threat–the burgeoning concern about the loss of power of the dominant American social group that represents a salient symptom of societal division. In line with Hofstadter’s description, we found strong relationships between perceived status threat and both conspiracy mentality and subclinical paranoia. Further, while there were those across the political spectrum who endorsed concerns with status threat, we replicated prior work showing an association between perceived status threat and political conservatism. Finally, we observed a small but significant positive association between perceived status threat and age. Critically, conspiracy mentality, paranoia, and conservatism were largely non-overlapping in their association with status threat, suggesting that they each represent a unique correlate of these concerns. Further, in contrast to past research, we observed no positive association between conservatism and subclinical paranoia (though conservatism and conspiracy mentality were modestly positively correlated, in line with past work [58]), highlighting the importance of using caution when operationalizing paranoia in sub- or non-clinical contexts.

As instances of racial violence [53] and major political upheaval [52] continue to bear the mark of elevated status threat concerns, understanding its psychological correlates represents an issue of increasing importance. Our findings may have implications for policies that attempt to reduce the impact of sources that spread exaggerated or false information regarding status threat. For example, media that exaggerates interpersonal threat from racial minorities (i.e., intersecting paranoia- and status-threat-relevant themes) and/or attributes status-threatening events to the clandestine actions of nefarious groups (i.e., intersecting conspiracy- and status-threat-relevant themes) may be particularly important to “prebunk” [59] or target using “nudge” interventions [60]. To this end, future research could test interventions that address exaggerated status threat concerns, clarify causal pathways between relationships described herein, and explore related factors that may contribute to perceived status threat or otherwise interact with these variables (e.g., distrust in institutions).

## Supporting information

S1 File Preregistered analyses and sample demographics. (DOCX)
