# Supplementary material for: Conspiracy mentality, subclinical paranoia, and political conservatism are associated with perceived status threat
Source: PLoS One. 2023 Nov 22;18(11):e0293930. doi: 10.1371/journal.pone.0293930 (PMC10664880; doi:10.1371/journal.pone.0293930)
Supplement: S1 File — (DOCX) [file pone.0293930.s001.docx]

**Supporting Information**

**Table of Contents**

| **Section** | **Title** |
| --- | --- |
| **Section 1** | **Preregistered analyses** |
| **Section 2** | **Sample demographics** |

**Section 1 – Preregistered analyses.**

The data presented in the main manuscript was collected in conjunction with a preregistered data collection and analysis plan (https://aspredicted.org/blind.php?x=G75_1J8). Here, we report the results of these preregistered analyses, in which we predicted that perceived status threat would moderate the relationship between extreme political beliefs (agnostic of party) and paranoia/magical thinking. More specifically, we anticipated that a positive relationship between political extremism and paranoia/magical thinking would be heightened at higher levels of perceived status threat. Additionally, we hypothesized that perceived status threat would similarly moderate the strength of the association between extreme political beliefs and endorsement of conspiracy theories.

Paranoia, magical ideation, conspiratorial thinking, and perceived status threat were measured using methods described in the main manuscript. Political extremism was indexed via a four-point scale that ranged from 0 to 3, with 0 corresponding to participants who identified as more moderate and 3 corresponding to participants who identified as either “Very Liberal” or “Very Conservative”. Analyses were conducted in R. Outliers in non-skewed data were defined as points greater than 3 *SD*s from the sample mean; outliers in skewed data were defined using methods outlined by Hubert and Van der Veeken (2008), as implemented by the RobustBase package (Todorov & Filzmoser, 2010). No outliers were detected in the outcomes of interest (paranoia, magical ideation, conspiratorial thinking).

To test our preregistered hypotheses, we ran three linear regression models. The first assessed paranoia (via R-GPTS-B score) as a function of the interaction between political extremism and perceived status threat. A significant regression equation was found, *F*(3, 296)=9.68, *p*<.001, *R*^2^_Adj._=0.08. However, contrary to our hypotheses, there was neither a significant main effect of extremism (*p*=.71) nor an interaction between extremism and status threat (*p*=.56). This model revealed a significant main effect of status threat, *ß*=0.25, 95% CI [0.06, 0.43], *p*=.026, such that participants who endorsed higher levels of status threat also tended to report greater degrees of paranoia (consistent with the relationship described in the main manuscript). The second linear regression model assessed magical ideation (via MIS score) as a function of the interaction between political extremism and perceived status threat. A significant regression equation was found, *F*(3, 296)=5.80, *p*<.001, *R*^2^_Adj._=0.05. Again, contrary to our hypotheses, there was neither a significant main effect of extremism (*p*=.61) nor an interaction between extremism and status threat (*p*=.69). No significant main effect of status threat was observed (*p*=.09). Finally, a third linear regression model assessed conspiratorial thinking (via CMQ score) as a function of the interaction between political extremism and perceived status threat. A significant regression equation was found, *F*(3, 296)=24.29, *p*<.001, *R*^2^_Adj._=0.19. Once again, contrary to our hypotheses, there was neither a significant main effect of extremism (*p*=.31) nor an interaction between extremism and status threat (*p*=.32). However, this model revealed a significant main effect of status threat, *ß*=0.35, 95% CI [0.05, 0.65], *p*<.001, such that participants who endorsed higher levels of status threat also tended to report greater degrees of conspiratorial thinking (consistent with the relationship reported in the main manuscript).

Together, while these analyses indicate interrelationships between status threat and both paranoia and conspiratorial thinking (as characterized in more detail in the main manuscript), they suggest that status threat *does not* differentially heighten paranoia, magical ideation, or conspiratorial thinking among those who endorse more extreme political beliefs.

**Section 2 - Sample demographics.**

Table 1

*Sample Demographics*

| **Gender**  Cisgender Man  Cisgender Woman  Transgender Woman  Non-binary | 145  153  1  1 |
| --- | --- |
| **Education**  Less than a high school degree  High school graduate (including G.E.D.)  Some college but no degree  Associate’s degree  Bachelor’s degree  Master’s degree  Doctoral degree  Professional degree | 2  32  74  26  106  46  8  6 |
| **Race**  Multiracial  White  Black/African American  Asian  Native American or Alaska Native  Other | 15  205  44  25  1  10 |
| **Ethnicity**  Spanish, Hispanic, or Latino  Not Spanish, Hispanic, or Latino | 17  283 |
| **Political Affiliation**  1 - Extremely Liberal  2 - Somewhat Liberal  3 - Slightly Liberal  4 - Moderate  5 - Slightly Conservative  6 - Somewhat Conservative  7 - Extremely Conservative | 64  71  42  45  29  26  23 |
| **Annual Total Household Income**  <$50,000  <$100,000  <$150,000  <$200,000  <$250,000  $250,000+ | 131  106  36  17  1  7 |
| **Age** | 45.01 (*SD* = 15.87) |
